# Microcystins and Reproductive Dysfunction: Mechanisms and Consequences

**DOI:** 10.3390/toxins18070281

**Published:** 2026-06-25

**Authors:** Zhixin Chen, Zhihan Shi, Ziyu Chai, Jiayue Su, Xueqiong Yao

**Affiliations:** 1 School of Public Health, Hengyang Medical School, University of South China, Hengyang 421009, China; 20235800204@stu.usc.edu.cn (Z.C.); 20225800207@stu.usc.edu.cn (Z.S.); 20235800132@stu.usc.edu.cn (Z.C.); 20242014211223@stu.usc.edu.cn (J.S.); 2 School of Basic Medical Sciences, Hengyang Medical School, University of South China, Hengyang 421009, China; 3 Laboratory of Ecological Environment and Critical Human Diseases Prevention of Hunan Province, School of Basic Medical Sciences, Hengyang Medical School, University of South China, Hengyang 421009, China

**Keywords:** microcystins, reproductive toxicity, transgenerational effects

## Abstract

Accelerating eutrophication of aquatic ecosystems worldwide has increased concern regarding cyanotoxin exposure as an emerging environmental and public health issue, with Microcystin-LR (MC-LR) among the most extensively studied congeners due to its widespread occurrence and high toxicity. Evidence from experimental animal and cellular studies indicates that MC-LR elicits pronounced toxic impacts on both the male and female reproductive systems. In males, MC-LR induces overt testicular injury, compromises the structural and functional integrity of the blood–testis barrier, and triggers severe disorders in reproductive hormone synthesis and secretion. In females, it precipitates ovarian dysfunction, impedes normal follicular maturation and development, and induces distinct embryotoxic effects. The underlying pathogenic mechanisms involve the synergistic interplay of multiple signaling pathways, primarily including oxidative stress induction, aberrant apoptosis activation, endocrine disruption, and epigenetic modifications. Of particular significance, emerging evidence suggests that parental exposure to MC-LR may induce intergenerational or potentially transgenerational reproductive effects through epigenetic modifications in germ cells, impairing fertility and developmental outcomes in subsequent offspring and thus posing a sustained, long-term threat to population-level health. This review systematically delineates the reproductive toxicity profiles and underlying molecular mechanisms of MC-LR, evaluates its transgenerational health hazards, and aims to furnish robust scientific evidence for the formulation of targeted environmental health policies and risk management strategies.

## 1. Introduction

The increasing severity of cyanobacterial blooms, driven by water eutrophication, represents an increasing global environmental challenge [1]. These blooms not only disrupt aquatic ecosystem stability but also release harmful toxins upon cell lysis. Among these, Microcystins (MCs)—the most prevalent class of cyanobacterial toxins—have become ubiquitous contaminants in diverse freshwater bodies, including drinking water sources [2]. Owing to their stable cyclic heptapeptide structure, MCs are resilient to heat, chemicals, and varying pH, that their stable cyclic heptapeptide structure contributes to environmental persistence and may reduce removal efficiency in some conventional water treatment processes [3]. While at least 279 variations of microcystin are known, microcystin-LR (MC-LR) is among the most extensively studied and environmentally prevalent congeners because of its widespread occurrence and high toxicity [4,5]. Beyond directly poisoning aquatic organisms (e.g., fish and zooplankton) and thereby disrupting ecological balance, MC-LR may bioaccumulate through aquatic food webs and experimental evidence suggests potential multi-organ toxicity following exposure [6,7].

Numerous studies have reported that MC-LR exhibits potent hepatotoxicity, neurotoxicity, and immunosuppressive effects [8,9,10,11,12]. While these three toxic effects are well-established, the gonads have been identified as a primary target organ alongside the liver. Consequently, the reproductive toxicity of MC-LR has become a important research focus [13,14]. Given the particular vulnerability of the reproductive system to environmental pollutants, experimental studies indicate that MC-LR exposure may impair fertility, alter embryonic development, and potentially induce intergenerational or transgenerational effects, thereby jeopardizing both public health and ecological security. Consequently, elucidating the mechanisms of MC-LR’s reproductive toxicity is imperative for reliable environmental risk assessment and the development of targeted public health and ecological conservation strategies.

This review comprehensively synthesizes the mechanisms underlying MC-LR-induced reproductive toxicity, key evidence from experimental models, as well as its effects on reproductive health in experimental models, wildlife species, and available human evidence.

## 2. Pathological and Reproductive Toxicity Manifestations Induced by MC-LR

### 2.1. MC-LR-Induced Male Reproductive Toxicity: Targeted Effects on the Male Reproductive System

#### 2.1.1. Sperm and Fertility Outcomes

MC-LR exposure has been associated with reductions in sperm quality, including decreased sperm count and motility and increased sperm abnormalities, evidenced by reduced sperm count and motility, along with an elevated incidence of sperm malformations [15].

#### 2.1.2. Histopathological Changes

Histopathological alterations have been reported in testicular tissues, marked by a disorganized spermatogenic cell arrangement of spermatogenic cells, increased cellular sloughing, and prominent chromatin/nuclear condensation within the seminiferous epithelium [16,17]. The testes displayed significant atrophy, manifested by a reduced testicular organ coefficient (testis weight relative to body weight). This was accompanied by marked degeneration of the seminiferous tubules, characterized by a decrease in tubular diameter, a reduction in epithelial thickness, and expanded areas of interstitial fibrosis [17].

#### 2.1.3. Cellular and Molecular Mechanisms

Experimental studies have reported that MC-LR may activate Gasdermin D (GSDMD)-mediated pyroptotic pathways, a key effector protein involved in pyroptosis, leading to membrane pore formation. This triggers the release of proinflammatory factors (e.g., IL-1β), which disrupts the blood–testis barrier microenvironment. Concurrently, mitochondrial integrity is compromised, evidenced by swelling, ruptured cristae, diminished membrane potential, and impaired ATP production in spermatogenic cells, which may contribute to activation of Caspase-9-dependent apoptotic signaling [16,17,18].

#### 2.1.4. Endocrine Effects

At the hormonal level, evidence suggests that MC-LR may impair testosterone synthesis through both direct and indirect mechanisms. Directly, it downregulates key steroidogenic proteins (StAR and CYP17A1), inhibiting the enzymatic conversion of cholesterol to testosterone. Indirectly, evidence suggests that MC-LR may interfere with hypothalamic–pituitary–testicular (HPT) axis regulation by suppressing hypothalamic Gonadotropin-releasing hormone (GnRH) release, thereby reducing pituitary LH secretion [19,20].

### 2.2. MC-LR-Induced Female Reproductive Toxicity: Targeted Effects on the Reproductive System

Experimental studies suggest that MC-LR exposure may adversely affect the female reproductive system at multiple levels.

#### 2.2.1. Oocyte-Level Effects

In oocytes, the damage is manifested as cytoplasmic vacuolation [21]. Additionally, MC-LR exposure has been associated with organelle-level alterations in oocytes, provoking abnormal spindle assembly and chromosome misalignment [21].

#### 2.2.2. Follicular and Ovarian Tissue Effects

At the tissue level, this also involves the disruption of oocyte–granulosa cell communication, which subsequently causes premature detachment from the follicular wall [21,22]. Follicular development is significantly impaired, characterized by a reduction in the transition from preantral to mature follicles and downregulation of the proliferation marker Ki-67 in granulosa cells [23]. Studies have reported that MC-LR exposure induced significant pathological damage in ovarian tissue. This damage was characterized by enlarged intercellular spaces, detachment of follicular cells, and vacuolation of parenchymal cells [24].

#### 2.2.3. Molecular Mechanisms

Furthermore, experimental findings suggest that these effects may be associated with increased extracellular signal-regulated kinase (ERK) phosphorylation in ovarian tissue [24]. MC-LR exposure has been associated with organelle-level alterations in oocytes, provoking abnormal spindle assembly, chromosome misalignment, and a decline in mitochondrial membrane potential (MMP) [21].

#### 2.2.4. Reproductive Consequences

Ultimately, these collective alterations are consequently potentially contributing to ovulatory dysfunction and a reduced ovulation rate [23], thereby potentially compromising oocyte quality and contributing to adverse reproductive and developmental outcomes in experimental models [25].

### 2.3. Intergenerational and Developmental Toxicity of MC-LR in Offspring

Parental exposure to experimental evidence suggests that MC-LR exposure may induce epigenetic alterations in germ cells that could contribute to intergenerational or potentially true transgenerational effects, which underlies the developmental and reproductive dysfunctions observed in subsequent generations. Previous studies have found that in zebrafish and rat testes exposed to MC-LR, the level of MC-LR exposure has been associated with dose-dependent alterations in DNA methylation patterns in zebrafish and rodent models [26]. These epigenetic modifications were heritably transmitted via sperm to the F1 generation, representing an intergenerational effect which may contribute to altered Activated Protein Kinase (MAPK) signaling and changes in the expression of neurodevelopment-related genes (e.g., bdnf, psd95, gfap) [27]. Consequently, this led to impaired locomotor activity and aberrant brain ultrastructure in the offspring [28]. MC-LR exposure has been associated with altered Hsp90α expression and disrupted piRNA regulation, which disrupts piRNA biosynthesis and induces epigenetic silencing in sperm. This paternally inherited silencing, particularly of piRNAs regulating implantation and lung development, resulted in intergenerational abnormalities in F1 offspring with abnormal lung structure and dysregulated Wnt/β-catenin signaling [29]. Furthermore, a broader impairment of reproductive function was observed across consecutive generations: F1 offspring showed delayed reproduction and reduced fertility, while the unexposed F2 generation exhibited further declines in both survival and reproductive capacity, demonstrating that MC-LR induces true transgenerational inheritance through gametic epigenetic memory [26]. Most evidence for these effects currently derives from experimental animal models, and the relevance to human reproductive health requires further investigation. Collectively, these cross-species studies (from frogs, humans, zebrafish, and mice) indicate that MC-LR-induced reproductive toxicity is a conserved phenomenon, although the underlying mechanisms may vary among species. To better illustrate the studies on the relationship between MC-LR exposure andre-productive toxicity, we provide Table 1 below.

## 3. Mechanisms of MC-LR-Induced Reproductive Toxicity

### 3.1. Primary Molecular Initiating Event: MC-LR Uptake and PP1/PP2A Inhibition

The toxicity cascade of MC-LR is triggered by specific cellular uptake, followed immediately by a well-established primary molecular initiating event. Protein phosphatases (PPPs) are critical negative regulators of phospho-signaling, catalyzing the removal of phosphate groups from specific substrates to control pathway activity. Within this family, PP1 and PP2A constitute the principal subclasses, collectively representing the vast majority of phosphatase activity in eukaryotic cells [30]. The specific binding of MC-LR to PP1/PP2A catalytic subunits serves as the experimentally established primary molecular initiating event. This inhibition leads to a critical dysregulation of phosphorylation dynamics in germ cells and the concomitant activation of downstream apoptotic signals, resulting in programmed cell death [31].

### 3.2. Secondary Cellular Responses: Oxidative Stress, Mitochondrial Dysfunction, and ER Stress

Following the primary inhibition of PP1/PP2A, affected cells exhibit profound secondary stress responses, fundamentally characterized by oxidative stress, mitochondrial damage, and endoplasmic reticulum (ER) stress. MC-LR synergistically induces oxidative stress by depleting the antioxidant defense system (e.g., SOD, CAT, GPx) and simultaneously promoting Reactive Oxygen Species (ROS) production via NADPH oxidase activation and mitochondrial dysfunction [32,33]. ROS may contribute to MC-LR-induced germ cell apoptosis primarily via DNA damage and mitochondrial dysfunction [11,34]. MC-LR-induced oxidative stress is proposed to orchestrate mitochondrial fission by upregulating dynamin-related protein 1 (DRP1) through the transcription factor Forkhead box protein M1 (FOXM1), positioning DRP1 as a putative key effector of mitochondrial dysfunction [35]. Oxidative stress leads to sperm/oocyte impairment. Concurrently, MC-LR disrupts intracellular organelle homeostasis, leading to ER stress. MC-LR disrupts ER morphology, thereby inducing the accumulation of unfolded/misfolded proteins and triggering endoplasmic reticulum stress (ERS) [36]. By disrupting endoplasmic reticulum homeostasis, MC-LR triggers the unfolded protein response (UPR), which is hypothesized to act as a downstream mechanism of its germ cell toxicity. Notably, MC-LR activates the *PERK* pathway [37]. Upon activation, PERK phosphorylates eIF2α, thereby suppressing global protein synthesis as an adaptive mechanism to alleviate ER stress [38]. The role of MC-LR in triggering endoplasmic reticulum stress is evidenced by its activation of the PERK-eIF2α-ATF4 pathway and subsequent CHOP upregulation in diverse zebrafish tissues, such as the liver, ovary, and developing offspring [39]. Furthermore, its role in activating the IRE1α pathway has been established [40]. The activation of IRE1α leads to the cleavage and splicing of X-box binding protein 1 (XBP1) mRNA, generating the transcription factor sXBP1 [41]. This sXBP1 translocates into the nucleus, where it orchestrates the expression of a genetic program dedicated to restoring ER proteostasis through enhanced folding and degradation capacity [42]. ER stress leads to follicular degeneration. It should be noted that the studies discussed in this section are derived from diverse experimental models, including cell lines, pancreatic cancer cells, mouse ovaries, rats, and zebrafish. While these cross-model findings collectively support the reproductive toxicity of MC-LR, caution should be exercised when extrapolating results across different systems due to biological and physiological differences.

### 3.3. Tertiary Signaling Consequences: Apoptosis, Inflammation, and Endocrine Disruption

The accumulation of secondary cellular stress subsequently activates cascading tertiary signaling consequences, manifesting functionally as apoptosis, immune–inflammatory responses, and endocrine disruption.

Regarding apoptosis, It is hypothesized that ROS-triggered oxidative stress opens the mitochondrial permeability transition pore (PTP), thereby reducing the membrane potential and promoting cytochrome c (*Cyt c*) release. Cyt c complexes with Apaf-1 to form the apoptosome, activating Caspase-9, which in turn cleaves and activates Caspase-3/7 to execute apoptosis [33,43]. Similarly, prolonged ER stress drives apoptosis, this phospho-eIF2α-dependent translation enables the synthesis of transcription factor ATF4, which ultimately induces the expression of its downstream target, CHOP [44]. CHOP is a key factor in endoplasmic reticulum stress-induced apoptosis [44]. MC-LR has been shown to induce CHOP expression at both transcriptional and translational levels in mouse liver [45]. Additionally, activated IRE1α serves as a platform to recruit and activate downstream signaling molecules, including c-Jun N-terminal kinase (*JNK*). This cascade ultimately leads to NF-κB activation, thereby triggering inflammatory responses and apoptosis [42]. MC-LR accelerates follicular atresia in ovarian granulosa cells by inducing JNK-mediated damage to adherens junctions [46].

Parallel to apoptosis, MC-LR initiates a robust immune–inflammatory response. The reproductive toxicity of MC-LR is hypothesized to involve secondary coordinated multicellular immune–inflammatory responses with distinct cell-type specificity. In testicular tissue, MC-LR has been proposed to activate supporting cells (SCs), germ cells (GCs), and Leydig cells (LCs) via divergent pathways [47]. After directly entering SCs and GCs, MC-LR inhibits PP2A, leading to sustained PI3K/AKT/NF-κB activation and the upregulation of pro-inflammatory cytokines (TNF-α, IL-6, MCP-1, CXCL10). In LCs, which cannot internalize the toxin, inflammation is initiated via Toll-like receptor 2 (TLR2) activation, though the precise binding mechanism awaits elucidation [47,48]. Notably, SC-derived inflammation is amplified epigenetically; experimental evidence suggests that inflammatory signaling and microRNA-mediated regulation may contribute to reproductive toxicity by targeting MAPK11. This secreted TNF-α acts paracrinally on TNF receptor 1 (*TNFR1*) on GCs, triggering apoptosis [48]. In the ovary, MC-LR was mainly distributed in granulosa cells (mGCs), inducing mitochondrial DNA (mtDNA) leakage that activates the cGAS-STING pathway and amplifies *TNF-α* production [49]. These factors create a local inflammatory milieu and may stimulate stromal proliferation via paracrine action, forming a positive feedback loop [49]. Collectively, these findings suggest multiple interacting pathways contributing to reproductive toxicity wherein MC-LR induces cell-specific immune responses through direct enzyme inhibition, pattern recognition receptor activation, and epigenetic regulation. Inflammation leads to gonadal dysfunction.

Furthermore, these cellular disruptions culminate in severe endocrine-disrupting effects. The maturation of the follicle is characterized by a shift from gonadotropin independence (primordial to early secondary stages) to follicle stimulating hormone (FSH) dependence [50,51]. This hormonal regulation, commencing at the early antral stage, is indispensable for terminal follicular development and the acquisition of ovulatory potential [52]. Current evidence indicates that FSH-dependent follicular maturation requires the cAMP/PKA and PI3K/AKT/FOXO1 pathways [53,54]. MC-LR, following its established molecular initiating event of inhibiting PP2A, is hypothesized to disrupt the PI3K/AKT/FOXO1 signaling axis, thereby downregulating genes critical for follicular maturation and ultimately delaying oocyte maturation [42]. The reproductive toxicity of MC-LR involves the disruption of the hypothalamic-pituitary-gonadal (HPG) axis, which functions as the central regulator of reproduction. The axis operates through sequential signaling: GnRH from the hypothalamus prompts the pituitary to secrete FSH and Luteinizing Hormone (LH), which in turn stimulate gonadal production of 17β-estradiol and testosterone [55,56]. Current models suggest that MC-LR compromises this system by downregulating hypothalamic gnrh1/2 expression, leading to deficient FSH and LH signaling and ultimately impairing reproductive function [57]. Furthermore, gonadal development is modulated by growth hormone (GH). GH stimulates hepatic production of insulin-like growth factor (IGF), which directly acts on the gonads [58]. In a study by Hou et al., zebrafish exposed to 0.3, 3, or 30 μg/L MC-LR for 30 days exhibited suppressed GH transcription. This suppression of the GH/IGF axis consequently led to reduced IGF1/2 expression, ultimately resulting in delayed gonadal development [59]. Endocrine disruption leads to fertility effects.

### 3.4. Long-Term Consequences: Epigenetic Alterations and Developmental Effects

Ultimately, the cascade of primary, secondary, and tertiary events establishes long-term consequences, primarily characterized by epigenetic alterations and intergenerational impacts. Epigenetic mechanisms, including DNA methylation, histone modifications, and non-coding RNAs, critically regulate gene transcription [60]. Importantly, MC-LR exposure has been associated with altered DNA methylation and histone modification patterns in experimental models; these changes are generally locus-specific, tissue-specific, and dose-dependent, rather than universal genome-wide effects. A key example is histone acetylation, dynamically controlled by histone acetyltransferases (HATs) and histone deacetylases (HDACs). MC-LR disrupts this balance by upregulating HDAC1 and inhibiting HAT activity, leading to altered histone acetylation (reduced Ac-H3/Ac-H4). This compacts chromatin, thereby repressing the anti-apoptotic gene *Bcl-2* and activating pro-apoptotic genes (Bax, Caspase-3, Caspase-8), ultimately triggering the mitochondrial apoptosis pathway [43]. Concurrently, local histone deacetylation induces G1/S phase arrest by downregulating Cyclin D1 and upregulating p21Waf1/Cip1, thereby trapping cells in S phase and synergistically amplifying apoptosis [43]. At the DNA methylation level, MC-LR elevates DNMT expression, leading to altered DNA methylation patterns in sperm. A key example is the hypermethylation of the brain-derived neurotrophic factor (*BDNF*) promoter, which is proposed to disrupt paternal epigenetic transmission. Consistent with this, F1 male offspring exhibited increased BDNF promoter methylation, reduced BDNF expression, and a hypothesized secondary inhibition of the BDNF/AKT/CREB signaling pathway. Conversely, hypomethylation at promoters such as Dio3 and Gad1 may decouple methylation from transcription, resulting in aberrant gene expression in offspring [61]. In downstream signaling, hypermethylation of the BDNF promoter inhibits the BDNF-TrkB-PI3K/AKT-CREB axis, impairing neuronal proliferation and disrupting neurotransmitter homeostasis in offspring. Concurrently, MC-LR promotes apoptosis by enhancing H3K4 methylation to activate *p*53 transcription, while its inhibition of PP2A concurrently augments C-myc-mediated growth. This dichotomous regulation—hypermethylation of BDNF alongside hypomethylation of *Dio3*/*Gad1*—likely arises from the locus-specific targeting of DNMT/TET enzymes [61].

**Figure 1:**
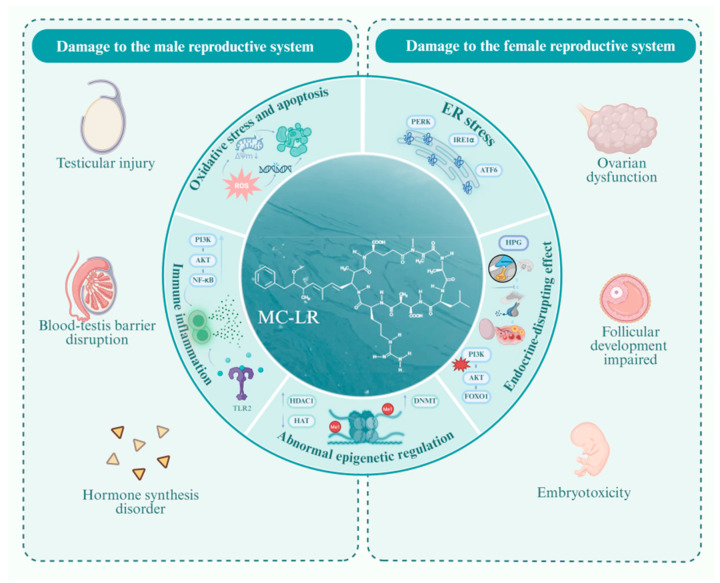
Mechanisms related to MC-LR-induced reproductive toxicity. Abbreviations: PI3K: Phosphoinositide 3-kinase. AKT: Protein kinase B. NF-Kβ: Nuclear factor kappa-B. PERK: Protein kinase R-like endoplasmic reticulum kinase. IRE1α: Inositol-requiring enzyme 1 alpha. ATF6: Activating transcription factor 6. HPG: Hypothalamic-pituitary-gonadal axis. FOXO1: Forkhead box protein O1. HDAC1: Histone deacetylase 1. DNMT: DNA methyltransferase. HAT: Histone acetyltransferase.

Figure 1 illustrates MC-LR-induced reproductive toxicity mechanisms.

## 4. Experimental and Epidemiological Evidence for MC-LR-Induced Reproductive Toxicity

### 4.1. In Vivo Experimental Models

Experimental models for assessing MC-LR reproductive toxicity are broadly divided into mammalian and non-mammalian systems. Mammalian models, especially rats and mice, are widely used owing to their short reproductive cycles, well-defined genetics, and physiological relevance to humans. Regarding exposure conditions, these models are typically subjected to chronic, low-dose oral or intraperitoneal administration to mimic environmental exposure scenarios. The principal findings from rodent models consistently provide evidence of severe reproductive outcomes, including significant sperm reduction, testicular/ovarian structural damage, and profound endocrine disruption. However, a notable limitation of rodent models is the potential for interspecies differences in toxicokinetics and susceptibility compared to humans. In contrast, non-mammalian models like zebrafish offer distinct advantages for developmental toxicity assessments due to external fertilization and embryonic transparency, allowing direct observation of morphological and functional changes during early development. Following aqueous exposure, major findings in zebrafish models include delayed hatching, gonadal dysgenesis, and distinct intergenerational developmental defects. Nevertheless, their primary limitation is the evolutionary distance, which restricts the direct translation of systemic and endocrine findings to mammalian physiology.

### 4.2. In Vitro Experimental Models

In vitro systems—including germ cell lines, primary cultures, and 3D organoids/co-cultures—are valuable for dissecting molecular mechanisms of MC-LR reproductive toxicity. Their primary strength lies in mechanistic precision, reproducibility, rapid throughput, and cost-effectiveness under controlled, direct-exposure conditions. Conversely, their major limitation is the lack of systemic interactions, toxicokinetic processing, and whole-body compensatory mechanisms. The following sections outline the applications and limitations of these models.

Monolayer germ cell lines facilitate the targeted analysis of toxicity mechanisms in specific germ cell types. For example, in mouse spermatogonial cells (GC-1 spg), MC-LR enters cells primarily through organic anion transporting polypeptides (OATPs), resulting in increased ROS production and DNA damage [34]. In bovine Sertoli cells (TM4), MC-LR activates Toll-like receptor 4 (TLR4), initiating downstream signaling cascades that lead to NF-κB activation and upregulated expression of pro-inflammatory cytokines including TNF-α, IL-1β, and IL-6 [62].

Primary germ cell cultures may better mimic the physiological microenvironment than immortalized cell lines. For instance, in bovine primary Sertoli cells, MC-LR downregulates connexin 43 (Cx43), a gap junction protein vital for Sertoli–germ cell communication, thereby disrupting intercellular junctions and increasing blood–testis barrier permeability [63,64]. Cross-species comparisons show that zebrafish primary spermatogonia are appeared less sensitive to MC-LR than mammalian cells, likely due to higher antioxidant capacity [13].

### 4.3. 3D Organoids and Co-Culture Systems

Three-dimensional organoids and co-culture systems replicate the spatial organization, cellular diversity, and microenvironmental interactions of reproductive tissues. Their key strength is providing improved physiological relevance over 2D cultures, bridging the gap between simple cellular models and complex in vivo systems. Testicular organoids—often composed of spermatogonial stem cells, Sertoli cells, and Leydig cells—self-assemble into seminiferous tubule-like structures with apicobasal polarity [65]. Similarly, 3D ovarian follicle models offer valuable tools for studying female reproductive toxicity [66]. Major findings from these advanced models have begun to elucidate complex tissue-level responses to MC-LR, such as localized immune–inflammatory crosstalk and dynamic barrier disruption. However, challenges remain in standardizing protocols, maintaining long-term culture stability, and relying on systems that are often functionally immature compared to adult gonads.

### 4.4. Population-Based Epidemiological Evidence

Limited epidemiological evidence suggests potential associations between environmental microcystin exposure and reproductive outcomes, although causal relationships remain uncertain. In males, some studies have reported associations that exposure with reduced sperm count, decreased motility, and altered hormone levels (e.g., FSH, inhibin B) [67]. One cross-sectional study found that higher MC levels in semen correlated significantly with poorer semen quality [67]. In females, experimental studies suggest that MC-LR exposure may affect ovarian function, oocyte maturation, and endocrine regulation, potentially compromising fertility [34]. Real-world human exposure to MC-LR occurs through multiple pathways, with drinking water representing the most direct route. Monitoring data from eutrophic drinking water reservoirs have revealed concerning MC-LR concentrations. For example, in Nanwan Reservoir (Xinyang City, China), which supplies municipal drinking water, the overall mean concentration of MC-LR in summer approached the WHO drinking water guideline of 1 μg/L [68]. Beyond drinking water, humans are also exposed through consumption of contaminated foods. Irrigation with cyanotoxin-contaminated water leads to MC accumulation in crops, as documented in Egyptian farmlands where irrigation water contained MC concentrations up to 93.7 μg/L, leading to detectable MCs in potato tubers (up to 1100 μg/kg fresh weight), spinach, and other vegetables. Importantly, the estimated daily intake through food consumption exceeded the WHO tolerable daily intake of 0.04 μg/kg bw/day [69]. Additional emerging exposure pathways include inhalation of aerosolized toxins during recreational activities near cyanobacterial blooms, consumption of algal dietary supplements, and occupational exposure of water treatment plant workers and fishermen [70].

Despite these emerging findings, current epidemiological evidence on MC-LR reproductive toxicity in humans remains severely limited and subject to major uncertainties. First, most existing studies are cross-sectional, which cannot establish causality or exclude reverse causation. Second, exposure assessment is often based on single time-point serum or semen MC measurements, which may not reflect long-term or cumulative exposure patterns. Third, sample sizes in reproductive health studies remain small, limiting statistical power. Fourth, it is extremely challenging to isolate the effects of MC-LR from co-occurring environmental contaminants (e.g., heavy metals, microplastics, pesticides) that may act synergistically. Fifth, the generalizability of findings is uncertain given that most studies to date have been conducted in specific geographic regions in China, and the applicability to other populations with different exposure patterns and genetic backgrounds remains unknown. These limitations underscore the urgent need for well-designed longitudinal cohort studies with improved exposure assessment (e.g., repeated biomonitoring, integration of environmental monitoring data) to better characterize the potential reproductive health risks of chronic low-dose MC-LR exposure in humans.

## 5. Critical Controversies and Challenges

Major uncertainties and critical controversies persist in the study of MC-LR-induced reproductive toxicity. A substantial gap exists between acute high-dose laboratory conditions and environmentally relevant chronic low-dose exposure. Specifically, many experimental studies employ concentrations far exceeding environmentally relevant levels, potentially limiting extrapolation to ecological and human risk [3,70]. The lack of standardized exposure protocols and variability in exposure routes (e.g., oral, intraperitoneal, aquatic) introduce methodological inconsistencies, frequently leading to contradictory findings when comparing environmental versus experimental concentrations, even though toxicity exhibits clear dose- and time-dependence [3,4,13]. Furthermore, unresolved debates remain regarding mixture versus single-compound toxicity. Environmental contaminants such as heavy metals, pharmaceuticals, pesticides, nitrite, and microplastics often coexist with MC-LR and may exacerbate gonadal injury, complicating regulatory risk assessment [5,6]. Interspecies differences in metabolism, reproductive physiology, and target-organ sensitivity limit the extrapolation of animal data to humans [5]. Human epidemiological evidence remains sparse and is often constrained by small sample sizes, exposure assessment uncertainties, and complex confounding factors, further impeding precise regulatory risk assessments [13,67]. While oxidative stress, apoptosis, endoplasmic reticulum stress, and inflammation contribute to MC-LR-induced reproductive toxicity, and PP1/PP2A inhibition is recognized as a major molecular initiating event, downstream signaling interactions, pathway crosstalk, and cell-specific responses remain incompletely understood [5,13].

## 6. Conclusions

This review summarizes current evidence regarding potential mechanisms underlying MC-LR reproductive toxicity, offering critical insights for ecological and human health risk assessments of cyanotoxins and supporting the revision and optimization of environmental regulatory frameworks. Several important challenges remain prominent: the scarcity of long-term, low-dose environmental exposure data, limited elucidation of synergistic toxic effects in complex pollutant mixtures, and limited translational applicability between preclinical experimental models and human populations. Future research priorities should integrate real-time environmental monitoring, multi-omics profiling, and future research may benefit from well-designed longitudinal epidemiological studies and improved exposure assessment approaches, with a focus on adopting advanced predictive models such as reproductive organoids. Simultaneously, future studies may provide evidence supporting the refinement of drinking water guidelines and risk assessment frameworks for reproductive health outcomes. Addressing these challenges will improve the translation of experimental findings into more reliable ecological and human health risk assessments.

## 7. Methods

To identify relevant studies on the reproductive toxicity of microcystin-LR (MC-LR), we performed a systematic literature search in PubMed, Web of Science, and Scopus from inception up to May 2026. The following search terms were used in various combinations: “microcystin-LR”, “MC-LR”, “reproductive toxicity”, “ovary”, “testis”, “sperm”, “oocyte”, “follicle”, “steroidogenesis”, “endocrine disruption”, “oxidative stress”, “apoptosis”, and “mitochondrial dysfunction”. We included original research articles (in vivo and in vitro studies) and peer-reviewed reviews published in English. Studies were considered eligible if they directly evaluated the effects of MC-LR on any aspect of male or female reproductive function in mammals (including rodents and humans) or other vertebrate models (e.g., zebrafish, frogs), as well as in reproductive cell lines (e.g., granulosa cells, Leydig cells, Sertoli cells). We excluded conference abstracts, non-English articles, and studies that only investigated other microcystin congeners without MC-LR.

## Figures and Tables

**Table 1:** Summary of studies on the relationship between MC-LR exposure and reproductive toxicity (*N* = 10, 2011 to 2025).

Subject	Exposure Conditions	Toxicity Manifestations	Reference
Male Wistar rats	1, 10 μg/kg; 50 days	Enlarged spaces between the seminiferous tubules, enlargement of the lumen of the seminiferous tubules, swollen mitochondria	[16]
Male SPF mice	1, 3.2, 10 μg/L; 3, 6 months	Sperm quality ↓, T ↓, loss and derangement of spermatogenic cells, enlargement of the lumen of the seminiferous tubules, thinning of the spermatogenic epithelium	[17]
Male SPF Balb/c mice	20 μg/kg; 7 days	GnRH ↓, GnRH mRNA ↓	[19]
Male ICR mice	20 μg/kg; 35 days	CYP11A1 ↓, CYP17A1 ↓, T ↓, StAR ↓, ROS ↑	[20]
Porcine oocytes	0, 20, 40 and 60 μM/L; 44 h	PP2A ↓, *p*53 ↑, BAX ↑, BCL2 ↓, apoptosis in porcine oocytes ↑	[21]
Female mice	0, 1, 10, 40 μg/L; 3, 6 months	Estrus ↓, stillbirth rate ↑, number of living pups per litter ↓, CAT ↓, SOD ↓	[23]
Female zebrafish	0, 1, 5, 20 μg/L; 30 days	Deformation rate of the offspring ↑, oocyte vacuolation, oocyte nuclear pyknosis, intercellular enlargement of oocytes	[24]
Adult P. nigromaculatus	1 μg/L; 14 d	F1 DNA methylation level ↑, egg weight ↓, egg diameter ↓, sperm deformities ↑	[26]
Zebrafish	0, 5, 20 μg/L; 6 weeks	tgf β1 genes hypomethylation, p-p38, p-Erk1/2, and pJNK proteins in zebrafish larvae ↑	[28]
Male Balb/c mice	1, 7.5, 15, or 30 μg/L; 6 months	Thickened alveolar wall and the deposition of collagen	[29]

SPF: Specific Pathogen Free, ICR: Institute of Cancer Research. An upward arrow means increased content or activity, while a downward arrow indicates decreased content or activity.

## Data Availability

No new data were created or analyzed in this study.

## Abbreviations

The following abbreviations are used in this manuscript:

ATF4	Activating transcription factor 4
ATF6	Activating transcription factor 6
BDNF	Brain-derived neurotrophic factor
cAMP	Cyclic adenosine monophosphate
CAT	Catalase
CHOP	C/EBP homologous protein
Cx43	Connexin 43
CYP17A1	Cytochrome P450 17A1
Cyt c	Cytochrome c
DNA	Deoxyribonucleic acid
DNMT	DNA methyltransferase
DRP1	Dynamin-related protein 1
eIF2α	Eukaryotic initiation factor 2α
ER	Endoplasmic reticulum
ERK	Extracellular signal-regulated kinase
ERS	Endoplasmic reticulum stress
FOXM1	Forkhead box protein M1
FSH	Follicle-stimulating hormone
GCs	Germ cells
GH	Growth hormone
GnRH	Gonadotropin-releasing hormone
GPx	Glutathione peroxidase
GSDMD	Gasdermin D
HATs	Histone acetyltransferases
HDACs	Histone deacetylases
HPG	Hypothalamic-pituitary-gonadal axis
HPT	Hypothalamic-pituitary-testicular axis
IGF	Insulin-like growth factor
IRE1	Inositol-requiring enzyme 1
JNK	c-Jun N-terminal kinase
LCs	Leydig cells
LH	Luteinizing hormone
MAPK	Mitogen-activated protein kinase
MC-LR	Microcystin-leucine arginine
MCs	Microcystins
mGCs	Mouse granulosa cells
MMP	Mitochondrial membrane potential
mtDNA	Mitochondrial DNA
NADPH	Nicotinamide adenine dinucleotide phosphate
NF-Kβ	Nuclear factor kappa-B
PERK	Protein kinase R-like endoplasmic reticulum kinase
PI3K	Phosphoinositide 3-kinase
PKA	Protein kinase A
PPPs	Protein phosphatases
PS-MPs	Polystyrene microplastics
PTP	Permeability transition pore
ROS	Reactive oxygen species
SCs	Supporting cells
SOD	Superoxide dismutase
StAR	Steroidogenic acute regulatory protein
TET	Ten-eleven translocation methylcytosine dioxygenase
TLR2	Toll-like receptor 2
TLR4	Toll-like receptor 4
TNFR1	Tumor necrosis factor receptor 1
UPR	Unfolded protein response
XBP1	X-box binding protein 1
